# Social deprivation in adolescence and the risk of depression in young adulthood: a longitudinal analysis of linked administrative data from Northern Ireland.

**DOI:** 10.23889/ijpds.v9i5.2607

**Published:** 2024-09-18

**Authors:** Stein Menting, Ronald McDowell, Enya Redican, Michael Rosato, Jamie Murphy, Gerard Leavey

**Affiliations:** 1 Ulster University; 2 Bamford Centre for Mental Health and Wellbeing

## Objective

Adolescence is a vulnerable period for onset of depression. Socioeconomic deprivation may elevate the risk of depression. We explored the risk of depression in early adulthood in relation to deprivation during adolescence.

## Approach

97,561 individuals aged 10-14 years were grouped at baseline (2010) using small-area indicator of area-level deprivation (measured in quintiles) and linked with yearly antidepressant prescriptions (2010–2021). A logistic random-intercept multilevel model estimated the longitudinal trajectory of yearly antidepressant prescriptions (2010-2021) between deprivation quintiles in 2010, adjusting for sex and age at baseline.

## Results

In 2010, the marginal probability of receiving antidepressants for an average young person differed little between those living in the most (0.0028: 95%CI=0.0025,0.0032) and least (0.0027: 0.0023,0.0032) deprived areas (p=0.75). By 2021, the marginal probability had increased more for those who lived in the most deprived areas in 2010 (0.191: 0.187,0.195) compared to those who lived in the least deprived areas in 2010 (0.140: 0.135,0.145)(p<0.001). Estimated antidepressant prescriptions were higher in females compared to males (Odds Ratio[OR]=3.15: 3.00,3.29) and increased per year of age at baseline (OR=1.26: 1.24,1.28).

## Conclusions

Growing up in more socially deprived areas is associated with an increase in antidepressant prescription risk during early adulthood. Improvements in administrative data access and linkage are needed to further explore these findings.

## Implications

Policy makers and data custodians should recognise the potential for administrative data in identifying social disadvantage and associated vulnerabilities in the general population, and take steps to make this data accessible to the research community.

